# Nonsequential double ionization with mid-infrared laser fields

**DOI:** 10.1038/srep37413

**Published:** 2016-11-18

**Authors:** Ying-Bin Li, Xu Wang, Ben-Hai Yu, Qing-Bin Tang, Guang-Hou Wang, Jian-Guo Wan

**Affiliations:** 1 National Laboratory of Solid State Microstructures, Collaborative Innovation Center of Advanced Microstructures and Department of Physics, Nanjing University, Nanjing 210093, China; 2 Deparment of Physics, Kansas State University, Manhattan, KS 66506, USA; 3 College of Physics and Electronic Engineering, Xinyang Normal University, Xinyang 464000, China

## Abstract

Using a full-dimensional Monte Carlo classical ensemble method, we present a theoretical study of atomic nonsequential double ionization (NSDI) with mid-infrared laser fields, and compare with results from near-infrared laser fields. Unlike single-electron strong-field processes, double ionization shows complex and unexpected interplays between the returning electron and its parent ion core. As a result of these interplays, NSDI for mid-IR fields is dominated by second-returning electron trajectories, instead of first-returning trajectories for near-IR fields. Some complex NSDI channels commonly happen with near-IR fields, such as the recollision-excitation-with-subsequent-ionization (RESI) channel, are virtually shut down by mid-IR fields. Besides, the final energies of the two electrons can be extremely unequal, leading to novel e-e momentum correlation spectra that can be measured experimentally.

Interaction between intense laser fields (10^13^–10^16^ W/cm^2^) and gas-phase atoms or molecules has led to many new physical phenomena, such as high harmonic generation (HHG)^1^,^2^, above-threshold ionization (ATI)^3^,^4^,^5^, nonsequential double ionization (NSDI)^6^, attosecond pulse generation^7^,^8^, etc. The core physical process underlying these phenomena is the so-called recollision process^9^,^10^,^11^: An emitted electron, after being accelerated in the laser field for a fraction of an optical cycle, can be driven back by the oscillating laser field and recollide with its parent ion core.

The most important laser parameter for the recollision process is the wavelength λ. Most work has been done with 800 nm, the fundamental wavelength of Ti:Sapphire laser systems. With the advancement of ultrafast laser technology, such as optical parametric chirped pulse amplification (OPCPA), the laser wavelength has been pushed longer into the mid-IR regime with λ > 3 μm^12^. These wavelengths are desirable in order to achieve high enough electron recollision energies, which scales as λ^2^, for the purpose of dynamic imaging of molecular structures^13^,^14^ or generating high energy broadband radiations^15^.

We notice that up to now not much attention has been paid to the process of NSDI with longer wavelengths, especially in the mid-IR regime^16^,^17^,^18^. NSDI with near-IR wavelengths has been known to provide an excellent, probably also the simplest, platform for the study of electron correlation effects^19^,^20^,^21^,^22^,^23^,^24^,^25^,^26^,^27^,^28^,^29^,^30^,^31^,^32^,^33^,^34^,^35^,^36^, which are the foundation for all materials. The questions that we were curious about and trying to answer in this paper include: What do recollision and NSDI processes look like with mid-IR laser fields? Is there any substantially new physics?

We will show how the obvious effect of a much higher recollision energy, which scales as λ^2^, leads to unexpected changes to the NSDI process and dynamics. For example, the electron trajectories that dominate NSDI with mid-IR laser fields are no longer the short first-returning ones. Longer second-returning trajectories may be more efficient in producing NSDI, although they usually have lower returning energies than the first-returning trajectories do. For another example, some complex NSDI channels that are common or even dominant with near-IR laser fields, such as the so-called recollision-excitation-with-subsequent-ionization (RESI) channel, are virtually shut down by the much higher recollision energy. Therefore although the fact of higher recollision energies is obvious for mid-IR laser fields, the impact of this fact on the resulting NSDI process is not, due to complex interplays between the returning electron and the remaining ion core. We will also show that the above-mentioned changes to the NSDI dynamics leave their footprints on the e-e momentum correlation spectra, which can be measured experimentally.

This paper is organized as follows. We first give a brief introduction to the classical ensemble method that we use to obtain the numerical results in the following Method section. Then we present our main results in the Numerical Results section, followed by more elaborate discussions in the Discussions section. Finally a Conclusion section will be given.

## Method

The method that we use in this paper is the classical ensemble method developed by Eberly and coworkers^37^, which has been widely used to describe strong-field double ionization processes qualitatively or quantita-tively^23^,^25^,^26^,^30^,^35^,^38^,^39^,^40^,^41^,^42^,^43^,^44^,^45^. Although atoms are fundamentally quantum mechanical systems and the interaction between an atom and an external laser field is described by the corresponding multi-electron time-dependent Schrödinger equation, the computational load is extremely demanding if not impossible^46^,^47^. The classical ensemble method provides a computationally efficient and physically intuitive approach to gain insights of strong-field double ionization problems.

Since the details of the classical ensemble method have been explained in numerous previous publications as cited above, here we only give a brief introduction to it. The general idea is to mimic a quantum mechanical wave function using an ensemble of classically modeled atoms. Before the laser pulse is turned on, an ensemble of classically modeled atoms is first populated within the classically allowed phase space for a total energy *E*_*tot*_=−1.23 a.u., which is set to be the negative sum of the first two ionization energies of the Xe atom (12.13 eV and 20.98 eV, respectively). *E*_*tot*_ has the following form (atomic units are used unless stated otherwise)


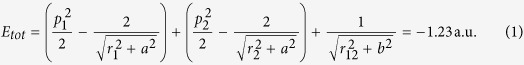


where 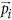 and 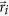 are the momentum and position of the *i*-th electron (*i* = 1, 2), 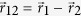
is the relative position of the two electrons, *a* and *b* are the soften parameters^48^,^49^ for the nuclear Coulomb potential and electron Coulomb potential. In our calculation we have set *a* = 2.0 a.u. to stabilize the classical atom (otherwise the classical atom will suffer from an unphysical autoionization effect), and *b* = 0.1 a.u. to prevent numerical singularity^45^. The size of the ensemble (i.e., the number of classically modeled atoms) is usually between 1 million and 10 million in our calculations.

After the initial microcanonical ensemble (i.e., every ensemble member has the same total energy) is populated, the laser electric field is turned on, and the two electrons start to move. The motions of the two electrons are governed by the time-dependent Newtonian equation of motion


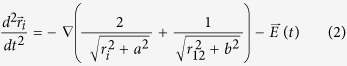


where 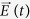 is the laser electric field. In our calculations here the laser field is polarized along the z direction and has a (2 + 6 + 2) trapezoidal shape, meaning that the laser pulse linearly turns on for 2 cycles, maintains full strength for 6 cycles, and then linearly turns off for 2 cycles.

The positions and momenta of the two electrons are recorded at each time step during the laser pulse. Statistics can be obtained for both experimentally measurable quantities, such as double ionization probability or end-of-pulse electron momentum correlation spectra, and theoretical quantities that cannot be measured experimentally, such as the emission times of the two electrons and the recollision time of the first-emitted electron. These theoretical quantities are very useful however to gain physical insights into the double ionization process.

## Numerical Results

### Probability of double ionization versus laser intensity

**Figure 1:**
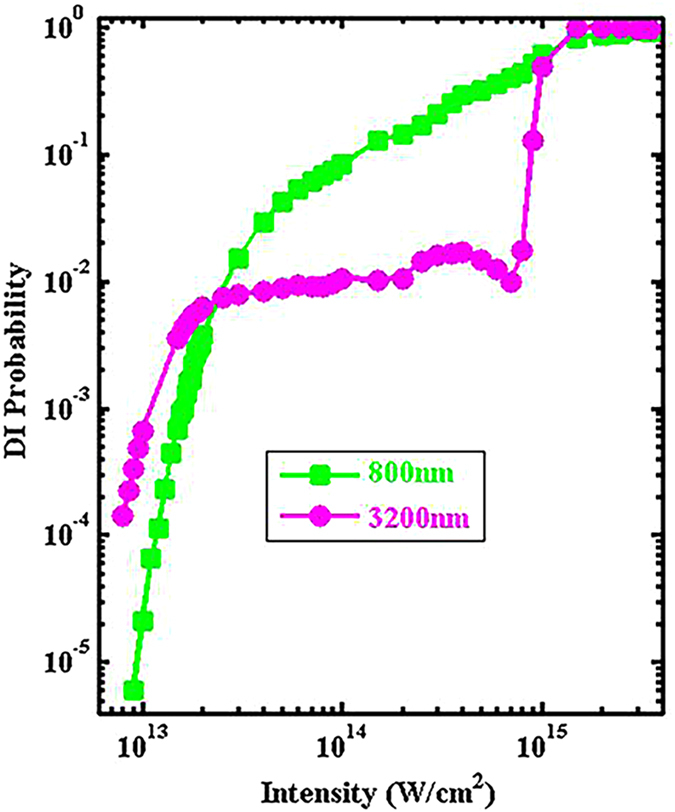
Probabilities of double ionization as a function of the laser peak intensity for the 800 nm (green squares) and 3200 nm (red circles) laser fields. The laser pulse has a trapezoidal shape with two cycles turning on, six cycles plateau, and two cycles turning off, for both wavelengths.

In this study we use two laser wavelengths, one is 800 nm (near-IR) and the other is 3200 nm (mid-IR). Figure 1 shows the probability of double ionization (DI) versus laser peak intensity for both the 800 nm (green squares) and the 3200 nm (red circles) laser fields. We can see that for both wavelengths, saturation of DI happens around 1.0 × 10^15^ W/cm^2^. Characteristic “knee” structures of NSDI can be seen in both cases below the saturation intensity.

It is interesting to see a crossing between the two curves around 2.0 × 10^13^ W/cm^2^, below which 3200 nm is more efficient in generating DI and above which 800 nm is more efficient. This is the result of competition between two factors: the returning energy and the returning flux (probability), of the first-emitted electron. The longer the wavelength, the higher the returning energy, the smaller the returning probability due to dispersion along the transverse direction (or wavepacket spreading along the transverse direction, in the quantum language, though a classical trajectory approach is used here). Below the crossing intensity around 2.0 × 10^13^ W/cm^2^, the returning energy wins, therefore the DI probability is higher for 3200 nm; above this crossing intensity, the returning energy is no longer a severe limitation for 800 nm in generating DI so the returning flux wins, therefore the DI probability is higher for 800 nm.

From this traditional DI probability versus laser intensity plot, we see that NSDI is the result of the interplay between the returning electron and the remaining ion core (which determines how much returning energy is needed to kick out the second electron). This interplay indeed lies at the heart of NSDI processes.

### Momentum correlation spectra of the two emitted electrons

**Figure 2:**
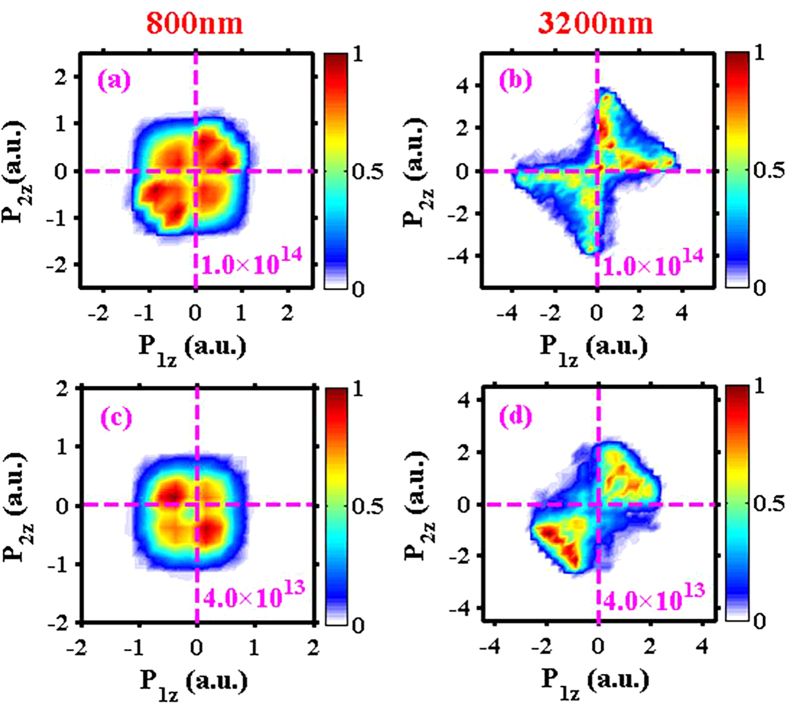
Electron momentum correlation spectra along the longitudinal direction. P_1z_ and P_2z_ denote the momentum components of the two emitted electrons along the laser polarization (longitudinal) direction, respectively. The data were not symmetrized. The upper row (**a,b**) is for laser intensity 1.0 × 10^14^ W/cm^2^ and the lower row (**c,d)** is for intensity 4.0 × 10^13^ W/cm^2^. The left column (**a,c**) is with 800 nm and the right column (**b,d**) is with 3200 nm.

Figure 2 shows the end-of-pulse momentum correlation between the two emitted electrons along the laser polarization direction (the z direction). For all the panels of this figure, the horizontal axis is the longitudinal momentum of one electron and the vertical axis is the longitudinal momentum of the other electron. The data were not symmetrized, so either electron could be the first-emitted electron. The left column (a, c) is for 800 nm and the right column (b, d) is for 3200 nm. The upper row (a, b) is for intensity 1.0 × 10^14^ W/cm^2^ and the lower row (c, d) is for intensity 4.0 × 10^13^ W/cm^2^, as labeled on each panel.

One can see that for the same laser intensity, the correlation spectra are drastically different for the two wavelengths. For the high intensity (upper row), for 800 nm slightly more electron pairs are correlated (i.e., population in the 1st/3rd quadrants) than anti-correlated (i.e., population in the 2nd/4th quadrants). The ratio between the two is about 57% to 43%. In contrast, for 3200 nm the majority (80%) of electron pairs are correlated. This change is even more dramatic for the low intensity case (lower row), with 46% correlated electron pairs for 800 nm and 83% for 3200 nm.

The shapes of the spectra are also completely different for the two wavelengths. Figure 2(a) shows a typical V-shape (or fingerlike) structure in the 1st/3rd quadrants^27^,^28^. The detailed mechanism leading to this kind of structure has been analyzed theoretically by Ye *et al*. in ref. 33. Changing the wavelength to 3200 nm, the spectrum shown in Fig. 2(b) features a cross-shape (X-shape) structure, with most populations concentrating along the axes, meaning that the final energies of the two emitted electrons are very unequal. Similar cross-shape structure has been reported experimentally in ref. 31 with however single-cycle near-IR laser fields and also known in field-free electron impact ionization^50^,^51^,^52^,^53^. The spectrum shown in Fig. 2(c), with more anti-correlated electron pairs than correlated ones, is a strong indication of the RESI double ionization channel^34^,^35^. Changing the wavelength to 3200 nm, however, Fig. 2(d) indicates that RESI channel is at least greatly suppressed because the spectrum is now dominated by correlated electron pairs.

### From first-returning to second-returning trajectories

More direct insight about the change of recollision dynamics from 800 nm to 3200 nm can be obtained from trajectory back analyses. We post-select all DI events and trace back the positions and the velocities of the two electrons as a function of time from the beginning to the end of the laser pulse.

For each DI event, we record the time of single ionization (t_SI_), the time of recollision (t_r_), and the time of DI (t_DI_). Here t_SI_ is defined as the time when the energy of the first-emitted electron (including the kinetic energy, the ion-electron potential energy and half of the e-e potential energy) just becomes positive, t_r_ is defined as the time of closest encounter between the two electrons after the departure of the first electron, and t_DI_ is defined as the time when the energies of both emitted electrons just become positive. Statistics of these characteristic times can be obtained after analyzing all DI events.

**Figure 3:**
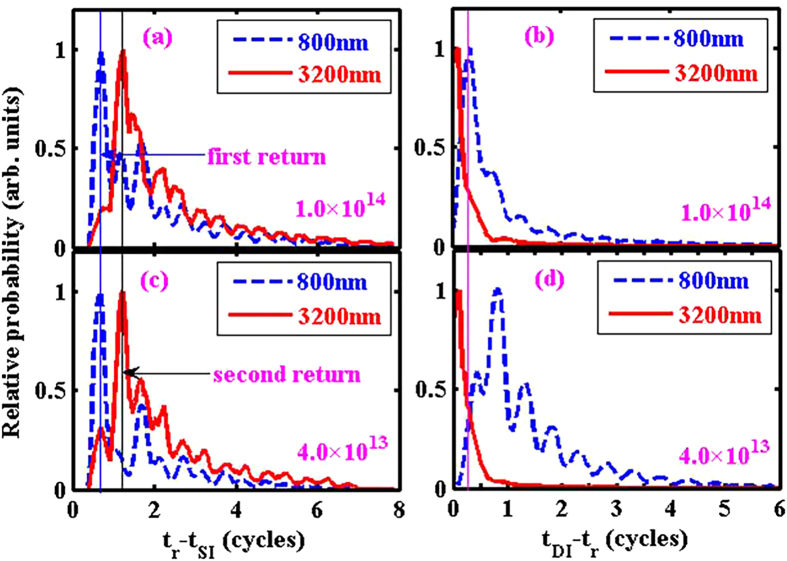
Left column (**a,c**): Statistical distributions of the time difference (t_r_ − t_SI_), i.e., the time difference between successful recollision and emission of the first electron, for two wavelengths and two laser intensities as labeled on the figure. Right column (**b,d**): Statistical difference of the time difference (t_DI_ − t_r_), i.e., the time difference between complete double ionization and recollision, for the same two wavelengths and laser intensities.

The left column (a, c) of Fig. 3 shows the statistical distribution of (t_r_ − t_SI_), the difference between the emission time and the later recollision time of e_1_. Figure 3(a) is for the high intensity case, with the blue dashed curve for 800 nm and the red solid curve for 3200 nm. One can see that the most probable value of (t_r_ − t_SI_) for 800 nm is peaked at about 0.75 cycles, which corresponds to the first-returning electron trajectories. For 3200 nm, however, the most probable value of (t_r_ − t_SI_) shifts to the peak at 1.25 cycles, which corresponds to the second-returning electron trajectories. The first-returning peak is greatly suppressed. Exactly the same trend appears for the low intensity case, as shown in Fig. 3(c). As will be explained later, this shift from first-returning to second-returning trajectories is the result of enhanced collision efficiency, or energy transfer between the two electrons during the recollision process.

### Suppression of the RESI channel

The time difference between complete DI and recollision, (t_DI_ − t_r_), is an indicator of NSDI mechanism. If this time difference is smaller than about 0.1 laser cycles, then the NSDI mechanism is recollision impact ionization (RII): the emission of the second electron is mainly due to the strong kick from the first electron. If this time difference is larger than about 0.25 cycles, then the NSDI mechanism is RESI: the first electron does not promptly kick out (though may excite) the second electron, and the second electron has to wait until the next field maximum to be pulled out by the laser field. Because recollision usually happens around field value zero, 0.25 cycles is the rough time to wait for the next field peak. Note however that there is no sharp and clear time cut to unambiguously distinguish different NSDI mechanisms.

The right column (b, d) of Fig. 3 shows the statistical distribution of this time difference (t_DI_ − t_r_) for 800 nm (dashed blue) and 3200 nm (solid red). The thin vertical line is the 0.25-cycles mark. One can see that for both intensities, for 3200 nm this time difference is very small with peak positions <0.1 laser cycles, indicating the prompt RII mechanism. In contrast, for 800 nm, this time difference peaks at 0.25 laser cycles for the higher intensity, and about 0.8 laser cycles for the lower intensity, indicating the dominance of the delayed RESI mechanism. Therefore changing the wavelength from 800 nm to 3200 nm, the RESI channel is greatly suppressed, leaving NSDI a simpler prompt RII process.

## Discussions

In the previous section we have shown numerical results that as the wavelength increases from 800 nm to 3200 nm, the most efficient electron trajectories to produce NSDI change from the shorter first-returning trajectories to the longer second-returning trajectories. Besides, the common or even dominant RESI channel for 800 nm is greatly suppressed for 3200 nm. These changes in NSDI mechanism will leave noticeable footprints on the experimentally measurable e-e momentum correlation spectra. In this section we discuss why these changes happen, as the wavelength increasing from 800 nm to 3200 nm.

### An example second-returning NSDI trajectory

**Figure 4:**
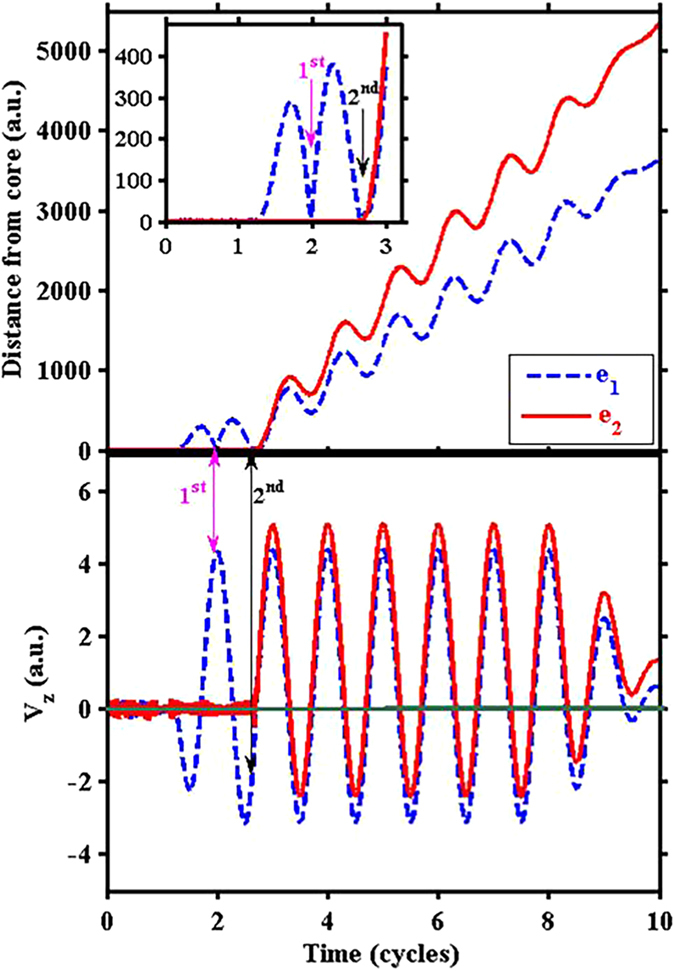
An example second-returning NSDI trajectory for 3200 nm with peak laser intensity 1.0 × 10^14^ W/cm^2^. Dashed blue curves are for the first-emitted electron (e_1_) and solid red curves are for the second-emitted electron (e_2_). The upper panel shows the distances of the two electrons from the ion core as a function of time during the laser pulse, and the lower panel shows the velocities of the two electrons as a function of time. The inset on the upper panel is a zoom in the early portion of the pulse, enlarging the two returning events.

To have an intuitive impression of second-returning electron trajectories for 3200 nm, we show an example in Fig. 4. The upper panel shows the distances of the two electrons from the ion core as a function of time during the pulse, and the lower panel shows the velocities of the two electrons along the z direction (i.e., polarization direction) as a function of time. The inset on the upper panel is a zoom in the initial portion of the trajectory, enlarging the two returning events. One can see that e_1_ was emitted around t_SI_ = 1.25 cycles when the laser field was around a peak and returned for the first time around t = 2.0 cycles when the laser field was about zero value. This first returning did not kick out e_2_. From the lower panel one can see that at the time of first returning, the velocity of e_1_ was about 4 a.u. Then e_1_ returned for the second time around t_r_ = 2.7 cycles and this time e_2_ was kicked out rather promptly. The velocity of e_1_ at the time of second returning was about 2 a.u., only half of the magnitude of that at the time of first returning, and in the opposite direction. Our statistics shows that about 52% of all NSDI trajectories are second-returning trajectories as exampled in Fig. 4, for the intensity 1.0 × 10^14^ W/cm^2^.

### The returning energy of the first electron and the ionization energy of the second electron

The above example NSDI trajectory shows that although e_1_ has a higher (4 times) returning energy at its first returning, it actually kicked out e_2_ at its second returning. This kind of first-returning-miss-and-second-returning-hit trajectories are not uncommon for 3200 nm and they constitute the statistical distributions of (t_r_ − t_SI_) shown in Fig. 3(a,c). From the intuitive Simpleman’s picture, it is also known that the energy of second returning is less than that of the first returning^54^. So the question to ask is why for 3200 nm the first returning trajectories with higher returning energies are less efficient in producing NSDI (i.e., kicking out the second electron) than the second returning trajectories with less returning energies.

To find the answer to this question we record the returning energies of each returning of the first-emitted electron for both 800 nm and 3200 nm. For 800 nm and 1.0 × 10^14^ W/cm^2^, the ensemble averaged first-returning energy is 0.66 a.u., and the ensemble averaged second-returning energy is 0.42 a.u. Both of them are less than the second ionization potential of Xe (i.e., the ionization potential of Xe^+^), which is 0.77 a.u. (20.98 eV). Therefore it is obvious to understand why under this wavelength and this intensity (or lower intensities) NSDI is dominated by the first-returning trajectories and the RESI mechanism.

In contrast, for 3200 nm and 1.0 × 10^14^ W/cm^2^, the ensemble averaged first-returning energy is 6.13 a.u., and the ensemble averaged second-returning energy is 3.13 a.u. Both of them are much larger than the second ionization potential of Xe. However, larger returning energy also corresponds to poorer recollision efficiency, because the returning electron is so fast that it spends little time in the vicinity of the ion core and little energy can be effectively transferred to the second electron. A reduced returning energy, as long as it is sufficient to kick out the second electron, has a higher energy transfer efficiency because the returning electron can spend more time interacting with the second electron. Of course, multiple-returning electron trajectories suffer from lower returning fluxes because of longer excursion time hence more spreading along the transverse direction. A balance can be achieved to maximize the NSDI efficiency. With the laser parameters that we are using, the optimal returning order to generate NSDI is the second returning.

For 3200 nm, even when the laser intensity reduces to 4.0 × 10^13^ W/cm^2^, the ensemble averaged second returning energy is 1.18 a.u., still larger than the second ionization potential. The ensemble averaged first returning energy is 3.05 a.u. Therefore even for this intensity, it is still the second-returning trajectories that lead to more efficient generation of NSDI.

### Extremely asymmetric energy sharing

As explained above, on the one hand mid-IR laser fields can produce returning electrons with high returning energies, much higher than the ionization potential of the second electron. On the other hand, because the first electron passes by the vicinity of the ion core very fast, the energy sharing between the two electrons is actually not as efficient as slower returnings with near-IR laser fields. These two factors combine and lead to extremely asymmetric energy sharing between the two electrons in the recollision process. The measureable effect is the cross-shaped e-e correlation spectrum shown in Fig. 2(b), with one electron having near-zero final momentum and the other electron having large final momentum.

**Figure 5:**
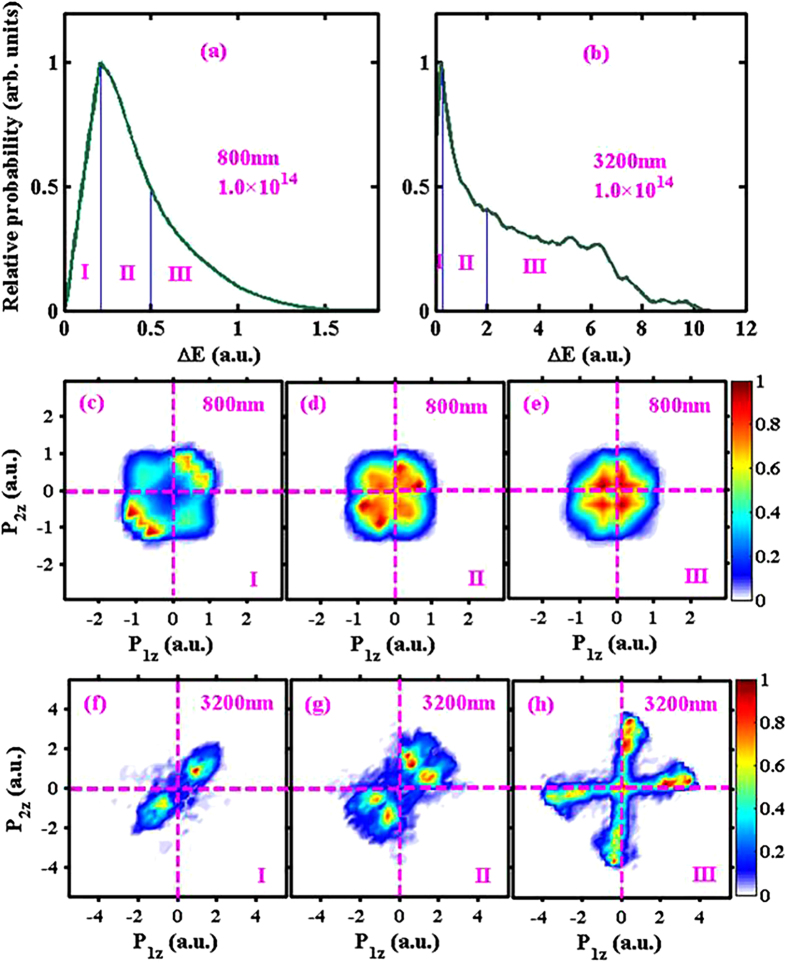
(Top row **a,b**) Distributions of Δ*E*, the absolute value of the energy difference between the two electrons right after (0.03 laser cycles after) each recollision, for 800 nm and 3200 nm. The intensity is 1.0 × 10^14^ W/cm^2^. The distributions are divided into three regions I, II, and III, quantifying the degree of energy sharing asymmetry. (Middle row **c,d,e**) End-of-pulse e-e momentum correlation spectra corresponding to the three regions of 800 nm, as labeled on each panel. (Bottom row **f,g,h**) End-of-pulse e-e momentum correlation spectra corresponding to the three regions of 3200 nm.

To illustrate this point in a more quantitative manner, we record the energies of the two electrons “right after” (e.g., 0.03 laser cycles after) each recollision. Then we take the (absolute value of the) difference between the energies of the two electron and plot the statistical distribution of this energy difference Δ*E*, as shown in Fig. 5(a) and (b) for 800 nm and 3200 nm, respectively. The intensity is 1.0 × 10^14^ W/cm^2^. This energy difference Δ*E*, taken right after each recollision, is an indicator of the degree of energy sharing between the two electrons during the just happened recollision process. The smaller the value Δ*E*, the more sufficient the energy sharing between the two electrons, vice versa. One can see that for 800 nm, Δ*E* extends to a maximum value of about 1.5 a.u., whereas for 3200 nm, Δ*E* extends to a maximum value of about 10 a.u. Besides, for 3200 nm there is an elevated plateau structure from about 2 a.u. to 6 a.u.

This asymmetric energy sharing between the two electrons during the recollision process has an obvious effect on the measurable end-of-pulse e-e momentum correlation, as shown in the lower two rows of Fig. 5. We divide the Δ*E* distributions into three parts (I, II, III) as illustrated in Fig. 5(a,b) and plot separately the e-e correlation spectra corresponding to these three parts. The division is somehow subjective but it helps to illustrate how different degrees of recollisional energy sharing affect the end-of-pulse e-e momentum correlation. From the lower two rows of Fig. 5, we see that e-e correlation spectra depend critically on the symmetry or efficiency of energy sharing, and the cross-shaped structure shown in the last panel (h) is indeed the result of extremely asymmetric energy sharing.

## Conclusion

In this paper we study how the recollision and NSDI processes will change when the laser wavelength increases from near-IR to mid-IR, using a well-established classical ensemble method. We show how the obvious effect of larger returning energies of the first-emitted electrons impacts NSDI in some unexpected and interesting ways. For example, with mid-IR laser fields the longer second-returning electron trajectories can be more efficient in producing NSDI than the shorter first-returning trajectories, although the returning energies are lower. For another example, mid-IR laser fields virtually shut down some complex NSDI channels which are common or even dominant with near-IR laser fields, such as the RESI channel.

We explain these unexpected results by considering the available returning energies of the first electron versus the ionization energy of the second electron. For near-IR laser fields, the available returning energies of the first electron are usually slightly higher or even lower than the ionization energy of the second electron, therefore the more energetic first-returning trajectories are favorable. For mid-IR laser fields, however, the available returning energies of the first electron are much higher than the ionization energy of the second electron. The slower second-returning trajectories can be more efficient in energy transferring during the recollision process, because the e-e interaction time is longer than the first-returning trajectories. This higher recollision efficiency compensates the lower fluxes of the second-returning trajectories due to more spreading along the transverse direction.

These changes in the NSDI mechanism will leave noticeable footprints on the experimentally measurable e-e momentum correlation spectra. For example, a cross-shaped structure with population concentrating on axes is likely to appear due to the extremely asymmetric energy sharing during the recollision process.

## Additional Information

**How to cite this article**: Li, Y.-B. *et al*. Nonsequential double ionization with mid-infrared laser fields. *Sci. Rep*. **6**, 37413; doi: 10.1038/srep37413 (2016).

**Publisher's note**: Springer Nature remains neutral with regard to jurisdictional claims in published maps and institutional affiliations.
